# Origin of apparent light-enhanced and negative capacitance in perovskite solar cells

**DOI:** 10.1038/s41467-019-09079-z

**Published:** 2019-04-05

**Authors:** Firouzeh Ebadi, Nima Taghavinia, Raheleh Mohammadpour, Anders Hagfeldt, Wolfgang Tress

**Affiliations:** 1 École Polytechnique Fédérale de Lausanne, Laboratory of Photomolecular Science, 1015 Lausanne, Switzerland; 2 Institute for Nanoscience and Nanotechnology, Sharif University of Technology, Tehran, 14588 Iran; 3 Department of Physics, Sharif University of Technology, Tehran, 14588 Iran

## Abstract

So-called negative capacitance seems to remain an obscure feature in the analysis of the frequency-dependent impedance of perovskite solar cells. It belongs to one of the puzzling peculiarities arising from the mixed ionic-electronic conductivity of this class of semiconductor. Here we show that apparently high capacitances in general (positive and negative) are not related to any capacitive feature in the sense of a corresponding charge accumulation. Instead, they are a natural consequence of slow transients mainly in forward current of the diode upon ion displacement when changing voltage. The transient current leads to a positive or negative ‘capacitance’ dependent on the sign of its gradient. The ‘capacitance’ appears so large because the associated resistance, when thinking of a resistor-capacitor element, results from another physical process, namely modified electronic charge injection and transport. Observable for a variety of devices, it is a rather universal phenomenon related to the hysteresis in the current–voltage curve.

## Abstract (web summary)

The apparent negative capacitance remains elusive in the impedance analysis of metal halide perovskite solar cells. Here Ebadi et al. show that it can be attributed to slow transients in the injection current instead of classical capacitive effect, i.e. charge accumulation.

## Introduction

Impedance spectroscopy (EIS) is a powerful technique to characterize semiconductor devices. It provides information regarding the (opto-) electrical (light, current, voltage) response on different timescales with high sensitivity. The measurement results are commonly analyzed with the aid of an equivalent circuit containing several passive elements. Assigning microscopic processes to these elements is generally the most challenging task. Nevertheless, this approach has turned out to be powerful, e.g., for the description of the physics of dye solar cells, where validated equivalent circuits have been established^1,2^. They can change depending on the applied large-signal voltage and become arbitrarily complex, in particular when distributions of states in energy (e.g. traps) or in space (non-constant electrochemical potentials) have to be considered, which can be done e.g. by introducing transmission line models.

Whereas resistive and capacitive elements can be directly related to transport, accumulation, and recombination of charges in common semiconductor devices, inductive features in the impedance are more puzzling. This is because they require the establishment of a considerable magnetic field, which is not present in the materials and geometries employed. As the impedance of an inductance is mathematically equivalent to an inverse and ‘negative capacitance’, the latter term has been introduced to describe an apparently inductive response. Based on the definition of capacitance (*C*), *C* = d*Q*/d*V*, a negative capacitance would correspond to a reduction of stored charge (*Q*) when the voltage (*V*) is increased. Such a situation can (theoretically) occur when an increased potential leads to a depopulation of interfacial states^3^ or under particular circumstances regarding time-dependent trap-assisted recombination in organic semiconductors^4^. The fact that negative capacitance is commonly observed for diodes under forward bias^5^ leads to conductivity modulations as a plausible explanation^6,7^. Such modulations can originate from self-heating, as reported for organic single-carrier devices^8^ and solar cells^9^.

In perovskite devices, negative capacitance at low frequencies has been reported already in early studies^10,11^. Although those studies proposed valuable explanations, they did not elaborate on them in detail, and confusion seems to remain. This is apparent from a recent study, where negative capacitance is found to have a deleterious effect on performance^12^. This conclusion was derived from a correlation of lower open-circuit voltage and fill factor with negative capacitance based on a comparison of two devices.

A feature related to negative capacitance is the inductive loop in the Nyquist plot, which corresponds to negative capacitance at intermediate frequencies. Such loops have been observed for a variety of devices, including those that contain SnO_2_ as an electron transport layer (ETL)^13^. Whereas that study (ref. ^13^) does not provide a specific explanation, another study where negative capacitance is claimed to occur only for ETL-free devices (and not for those containing SnO_2_), attempts an explanation using band diagrams^14^. In general, studies on negative capacitance in perovskites are so far rather speculative and there is a lack of a systematic approach to the origin of the effect. Only recently, theoretical studies started to elaborate more on this effect and propose out-of-phase recombination as a reason^15,16^. Therefore, despite the availability of established understanding for other material systems, a detailed study that clarifies negative capacitance in perovskite solar cells is urgently needed.

We show that negative capacitance in perovskite diodes is of the same nature as the slow positive response, which was originally ascribed to a ‘giant dielectric constant’^17^, and later to accumulation of electronic and ionic charge at interfaces^18^. However, we find that both positive and negative, low-frequency ‘capacitances’ do not describe charge accumulation, but are a consequence of transient injection currents modified by a slow process. The apparent capacitances are directly related to the scan-rate-dependent hysteresis^19^, both having in common that they describe the result of the effect rather than the effect itself. This interpretation relates to the coupled electronic-ionic impedance introduced by Pockett et al.^20^ and in that study attributed to ion-induced modified recombination rates.

## Results

### Large apparent negative and positive capacitances

**Fig. 1:**
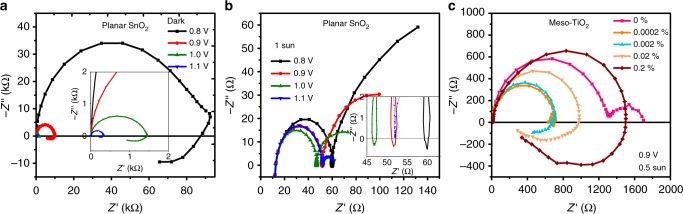
Nyquist plots for different perovskite solar cells, operated under forward bias. The frequency range is from 1 MHz to 0.1 Hz. **a**, **b** Planar device based on SnO_2_ at different voltages, where negative capacitance can be observed in the dark **a** and a loop feature under light **b**. **c** Mesoporous TiO_2_-based devices with more pronounced negative capacitance for higher bismuth content in the perovskite (in the legend given as at.% referred to Pb)

To start with, we present data of several devices in Fig. 1 (full data in Supplementary Fig. 1). These devices, reaching efficiencies up to 19%, employ a solar-cell architecture consisting of FTO/TiO_2_ (50 nm)/mesoporous TiO_2_ (150 nm)/perovskite (500 nm)/doped spiro-MeOTAD (150 nm)/Au (80 nm) with a nominal perovskite composition of Cs_0.1_FA_0.9_Pb(Br_0.1_I_0.9_)_3_. For planar devices, the TiO_2_ layers are replaced by a 15 nm thick SnO_2_ layer.

We confirm that negative capacitance is pronounced in planar devices with SnO_2_ as ETL^14^, where it emerges for forward bias, both in the dark (Fig. 1a) and under illumination, where inductive loops occur (Fig. 1b). However, negative capacitance can also be seen at stored devices based on mesoporous TiO_2_ (Supplementary Fig. 2). Furthermore, it can be provoked systematically by adding Bi as an impurity to the perovskite film (Fig. 1c). We observe that the negative capacitance feature becomes more pronounced with higher Bi content. This implies higher frequencies, where the reactance changes sign, and lower voltages, where the negative capacitance occurs (1–0.8 V, Supplementary Fig. 3). Bismuth is known to replace Pb in the lattice and causes a defect state leading to enhanced non-radiative recombination and therefore a reduction of the open-circuit voltage (*V*_oc_) (*JV* data in Supplementary Figs. 4 and 5)^21^. Only considering this Bi dataset, we could conclude that negative capacitance is related to enhanced recombination and a reduced *V*_oc_ (from 1.1 V to 0.9 V) as observed in ref. ^12^. However, the data of the SnO_2_ (*V*_oc_ = 1.1 V) or aged TiO_2_ (*V*_oc_ = 1.06 V) devices show also negative capacitance despite the high *V*_oc_, which is comparable to TiO_2_-based devices without negative capacitance. Therefore, a correlation of negative capacitance with a reduced *V*_oc_ is not necessarily given. Our data measured in the dark and under illumination opposes reports claiming that negative capacitance can only be observed under very specific conditions, e.g., regarding light intensity. Thus, the negative capacitance is a rather universal effect in perovskite devices similar to the inductive loop, where the negative capacitance feature is superimposed by a positive capacitance at low frequencies^13^. Our data suggest that negative capacitance might be related to hysteresis (Supplementary Fig. 2), which interestingly has not been discussed yet in the literature except for a very recent modeling study^15^. We will come back to it below.

**Fig. 2:**
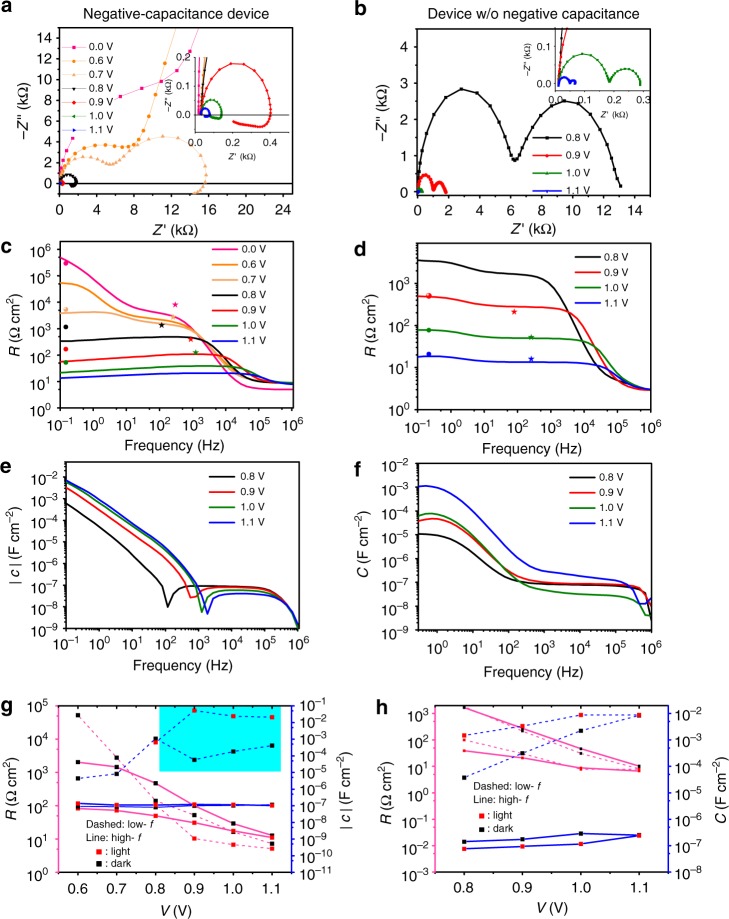
EIS data as a function of applied voltage in the dark. Left: negative-capacitance device (0.02% Bi). Right: device without negative capacitance (0% Bi). **a**, **b** Nyquist plots showing approximately two semi-circles and the appearance of negative capacitance for voltages larger than 0.7 V. **c**, **d** Real part of impedance, visualizing the electrode series resistance for frequencies >10^5^ Hz, a plateau at 1 kHz, and a higher (lower for negative *C*) low-*f* resistance. The symbols indicate the differential resistance obtained from *JV* scans with 10 mV s^−1^ (circles) and 10 V s^−1^ (stars). **e**, **f** Apparent capacitance $\frac{{{\mathrm{Im}}(1/Z)}}{\omega }$, where in **e** *C* values for frequencies lower than the one where the dip occurs are negative. **g**, **h** Resistance and capacitance (negative values shaded) obtained by describing the EIS data measured in the dark and under 1 sun by two *RC* elements in series. *R*_low *f*_ ∝ 1/*C*_low *f*_ is striking, in particular in **h**

For a more detailed analysis, we select one representative device with pronounced negative capacitance (ETL TiO_2_, perovskite with 0.02% Bi) and without (ETL TiO_2_, perovskite without Bi). Figure 2 shows different representations of the impedance *Z* at different applied bias in the dark. The Nyquist plots (Fig. 2a, b) show approximately two semi-circles in both cases, which can be represented by *RC* elements in an equivalent circuit. Their diameters are strongly decreasing for higher applied voltage indicative of a reduction of the resistance (*R*(*ω*) = Re(*Z*)) as seen in the plot of the real part of the impedance in Fig. 2c, d. For the negative-capacitance device (Fig. 2a), the low-frequency semi-circle transforms into the inductive region (−Im(*Z*) < 0) for higher voltages. This trend is independent of light intensity (Nyquist and capacitance plots for different light intensities in Supplementary Figs. 6 and 7). Correlated to the decrease of *R* is an increase in the capacitance function $\left( {C(\omega ) = \frac{{{\mathrm{Im}}(1/Z)}}{\omega }} \right)$ in the low-frequency regime (smaller than 100 Hz) shown in Fig. 2e, f. For the negative-capacitance device, the values of *C* for low frequencies (below 100 Hz to 1 kHz dependent on the voltage) are negative. Interpreting each semi-circle of the data in Fig. 2a, b as a *RC* element permits a reduction of the frequency-dependent representation of *R* and *C* to two pairs (*R*, *C*_high *f*_; *R*, *C*_low *f*_). Those are plotted as a function of voltage and for two illumination intensities (0 and 1 sun) in Fig. 2g, h.

We can readily assign a physical meaning to the high-frequency *RC*, such as a decreased *R*_high *f*_ with increased voltage due to enhanced conductivity of the diode. The approximately exponential decrease with voltage confirms expected changes in the differential resistance of a diode following the Shockley equation ($J = J_{\mathrm{S}}\left( {{\mathrm{exp}}\frac{V}{{nV_{\mathrm{T}}}} - 1} \right)$, with the saturation current *J*_S_, diode ideality factor *n*, and the thermal voltage *V*_T_). Illumination reduces the (phenomenological) resistance already at lower voltages as expected for solar cells with non-optimal fill factor, where the negative current under illumination decreases with increased voltage. This dependence of the current on voltage reduces the differential resistance $R = \left( {\frac{{{\mathrm{d}}J}}{{{\mathrm{d}}V}}} \right)^{ - 1}$. The rather unmodified *C*_high *f*_ in the order of 100 nF cm^−2^ describes the geometrical capacitance of the roughly 500 nm thick perovskite layer.

In contrast, the physics behind the low-*f RC* is not that obvious. This holds independent of whether *C*_low *f*_ is a negative or a positive apparent capacitance, because it is not a capacitance. Therefore, we do not need to postulate a space-charge accumulation as reason for the high (and photo-enhanced) capacitance as done in ref. ^18^. Instead of looking at *R*_low *f*_ and *C*_low *f*_ separately, we should focus on the time constant *τ*_low *f*_. In the following we will see that it remains constant independent of external factors (as observed for voltage in ref. ^22^) and is universal to all the devices investigated in this study. It is therefore indicative of a transient process that changes the device response with a time delay.

**Fig. 3:**
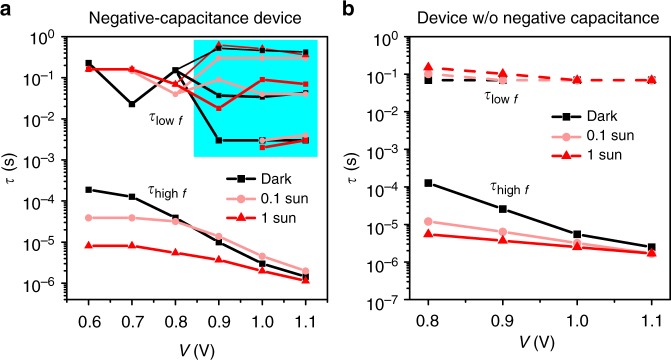
Time constants extracted from EIS data for different light intensities. **a** Negative-capacitance device. The capacitance changes sign at 0.8 V and the shaded area describes negative values, where the impedance data were fitted with multiple time constants. Details in Supplementary Table 1. **b** Device without negative capacitance. In both cases, the *τ*_low *f*_ remains independent of voltage and illumination at around 0.1 s. The data were extracted from the EIS data shown in Fig. 2

The time constants $({\tau = \frac{1}{{\omega |_{\max \left( {{\mathrm{Im}}\left( Z \right)} \right)}}} = RC})$ of each semi-circle are plotted in Fig. 3 as a function of voltage for three illumination intensities (0, 0.1, and 1 sun, Nyquist plots in Supplementary Figs. 6–8). First focusing on the device without negative capacitance (Fig. 3b), we observe that neither the applied voltage (as already observed in ref. ^22^) nor the illumination intensity have considerable effect on *τ*_low *f*_, which remains at approximately 0.1 s. This is in stark contrast to *τ*_high *f*_, which follows the trend in *R*_high *f*_. The negative-capacitance device behaves qualitatively the same. The transition from positive to negative capacitance between 0.7 and 0.8 V leads to slight variations. Furthermore, a range of time constants has to be given (shaded area) as obvious from the negative arc in the Nyquist plot (Fig. 2a) that cannot be described by a single semi-circle. A good fit has been obtained by considering three time constants, shown in the graph and summarized in Supplementary Table 1.

In general, the consequence of a constant *τ*, but reduced resistance is a higher apparent capacitance (*τ* = *RC*). Exactly this happens for the low-*f* contribution in perovskite devices. It explains directly all the observations related to an increase of capacitance under light^17,18,23,24^, which has, in fact, nothing to do with accumulation of photogenerated charges, as already pointed out for the positive *C*_low *f*_ in ref. ^20^. Furthermore, the matching of *τ*_low *f*_ for devices with and without negative capacitance is strong evidence for the fact that both processes are of the same origin as already hypothesized in ref. ^10^.

### Analysis in the time domain

**Fig. 4:**
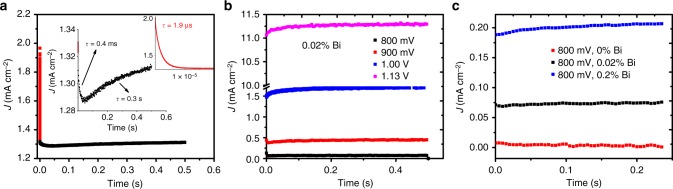
Transient current response upon applying a voltage step in the dark. **a** Negative-capacitance device at 0.9 V after applying +20 mV at time 0. Apart from fast capacitive responses, a transient increase of the current is observed that is the origin of the apparent negative capacitance. **b** Voltage dependence of the transients of a negative capacitance device. **c** Comparison of devices with differently pronounced negative capacitance

To better illustrate the meaning of these time constants, which result in the apparent capacitances, we discuss data for a ‘negative-capacitance’ device in the time (*t*) domain. In doing so we superimpose a small square voltage perturbation (20 mV) to a constant bias and record the transient current response on the oscilloscope (input impedance 50 Ω). An exemplary response for the device with negative capacitance at 0.9 V bias is shown in Fig. 4a, where we can identify three transient processes on different timescales. They fit roughly an exponential function as phenomenologically expected for an *RC* element, where application of the voltage perturbation leads to charging of the capacitor with a current: $J \propto \exp \left( { - \frac{t}{{RC}}} \right)$.

The two faster processes follow this equation with a positive value of *C*. The fast process with a time constant of roughly 2 μs is independent of voltage (Supplementary Fig. 9) and attributed to the *RC* response of the system (100 nF cm^−2^ × 0.27 cm^2^ × (50 + 10)Ω ≈ 2 μs, 50 Ω describe the input impedance and 10 Ω the electrode series resistance). The second transient (for times smaller than 1 ms) corresponds to the first semi-circle in frequency space (*τ*_high *f*_). The third transient, however, characterizes a rise in current instead of a decrease. When using the above equation, *C* naturally becomes negative. Comparing the time constant with *τ*_low *f*_ from the impedance data results in an excellent coincidence for all applied voltages (Supplementary Table 1). Note that analogous to the EIS data, the transient data cannot be reproduced with a single *τ*_low *f*_ to achieve a good quantitative fit. This further confirms that the representation by one *RC* element (with negative *C*) is not adequate. In devices without negative capacitance, this positive transient feature is not visible (Fig. 4c for a comparison and Supplementary Fig. 10 for a higher voltage).

The discussed representation in the time domain, although being equivalent to the frequency domain and transformable by Fourier analysis, is still beneficial because it facilitates distance of thought from the idea of a conventional capacitance somehow related to charge accumulation. Instead, it allows us to think of any transient process leading to a delayed increase of current when increasing the voltage as already described in ref. ^11^.

### Negative capacitance and hysteresis

**Fig. 5:**
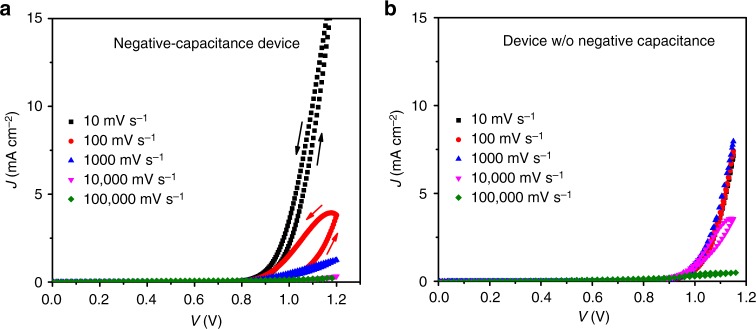
*JV* curves in the dark starting at 0 V for different scan rates. **a** Negative-capacitance device, **b** Device without negative capacitance. In case of negative capacitance, the dependence of forward current on scan rate is much more pronounced at lower scan rates

This way of thinking directly relates the negative capacitance effect to hysteresis in the current–voltage (*JV*) curve, analogously to the general low-frequency response of the impedance. To visualize this correlation, we measure the *JV* curve in the dark starting from 0 V and at different scan rates. The negative-capacitance device (Fig. 5a) indeed shows a pronounced dependence of forward current on scan rate, already becoming obvious between 10 and 100 mV s^−1^. The whole Bi series (displayed in Supplementary Fig. 11) shows that the more pronounced the negative capacitance, the stronger is the influence of the scan rate, and this effect already appears at low rates. The device without negative capacitance shows deviations from the steady-state curve only for very fast scans (scan rates higher than 10,000 mV s^−1^), where the positive capacitance (first semi-circle in the Nyquist plot) still dominates.

Consequently, devices with negative capacitance are characterized by a forward current that keeps slowly increasing after having applied a voltage. We will examine possible microscopic mechanisms for this modified current in the final part of this study. Independent of the exact mechanism, we conclude that similar to the hysteresis effect under light, which is not capacitive (despite claimed in ref. ^25^), but a response of the collection efficiency of photogenerated charges to modified electric fields in the device^26,27^, the high low-*f* capacitance (be it positive or negative) does not describe any process related to charge accumulation and cannot, therefore, be used to extract information on accumulated charges. Instead, it is the result of a modification of current, which can be referred to as conductivity modulation^6^. Our interpretation of a non-relation to accumulated charge is consistent with reports from other fields such as quantum-well infrared photodetectors^28^.

**Fig. 6:**
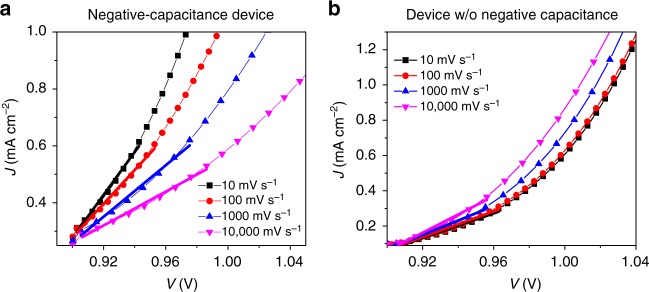
*JV* curves in the dark for different scan rates after equilibration at 0.9 V. **a** Negative-capacitance device, **b** Device without negative capacitance. The differential resistance is indicated by the lines. The trends in the resistance with scan rate are opposite between the two devices

We argued that *C*_low *f*_ does not have any meaning by itself. Now we want to investigate whether the associated resistance *R*_low *f*_ has a physical interpretation. From the data in Fig. 2 it becomes directly obvious that *R*_low *f*_ is correlated with *R*_high *f*_  —  an observation also reported in ref. ^22^ and in that study related to surface recombination. Following *R*_high *f*_, the *R*_low *f*_ decreases by orders of magnitude when the voltage is increased analogous to a reported dependence on light intensity^20^. Therefore, it is likely that it is related to the same physical process as *R*_high *f*_, which decreases with voltage because the diode turns on as discussed above. With Fig. 6 we discuss that indeed the two resistances describe the same phenomenon.

Figure 6a shows the *JV* curve obtained after preconditioning the negative capacitance device at 0.9 V for 20 s. The inverse differential slope at this point for a fast *JV* scan approximates *R*_high *f*_ of the impedance data. Whereas *R*_high *f*_ is often called recombination resistance, we prefer not to use this term, because it would imply that a reduced resistance would mean increased recombination, which one might relate to e.g. decreased *V*_oc_. However, this is not the case here. *R*_high *f*_ comprises all the contributions related to charge injection and transport as well. Coming back to Figure 6a, we observe that for slower scans, the current increases more rapidly with voltage consistent with the *JV* data from Fig. 5. The differential resistance decreases compared to the one for fast scans, as expected for the case of negative capacitance where the low-frequency resistance is smaller than the one at intermediate frequency. For a device with non-negative capacitance (Fig. 6b), the trend is inverted and much less pronounced, resulting in a larger resistance, which is expected for a second positive semi-circle. Quantified differential resistances at different voltages for low (10 mV s^−1^, circles) and high (10,000 mV s^−1^, stars) scan rates are added as symbols to the resistance plots in Fig. 2c, d. The frequency was chosen for conditions where the device behaved most resistive for the respective semi-circles in the Nyquist plots in Fig. 2a, b. The good coincidence shows that the resistance, including its complete frequency dependence can be described by a transient differential resistance of electronic charges.

## Discussion

We want to discuss a model for the microscopic process leading to the delayed current response. We summarize its characteristics: (i) Timescale on the order of seconds; (ii) Driven by voltage (AC in EIS, high scan rate in *JV*); (iii) Not dependent on the preconditioning voltage (DC in EIS); (iv) Not accelerated by illumination; (v) Changes the overall differential resistance; and (vi) Appears as a high low-*f* capacitance.

All these points, coinciding with the observations on hysteresis^19^, and a dependence of *τ*_low *f*_ on perovskite thickness^22^ indicate an ionic origin as commonly assumed in the literature. In particular, the independence of light intensity advocates against photogenerated electrons and holes as the source. The independence of bias voltage and therefore the position of electrochemical potentials is strong evidence against trap (distributions) dominating the behavior. Instead, it is consistent with defect ion migration^29^, whose dynamics do not seem to depend on light intensity in accordance with earlier reports^19^ indicating that the reported light-enhanced ionic conductivity^30^ is not present or at least without consequence in devices and on the timescales discussed here.

On the other hand, the resistances *R*_high *f*_ and *R*_low *f*_ describe processes related to electrons and holes. Those show a strong dependence on voltage and illumination intensity, because charge-carrier densities change by orders of magnitude upon varying these parameters in a solar cell.

**Fig. 7:**
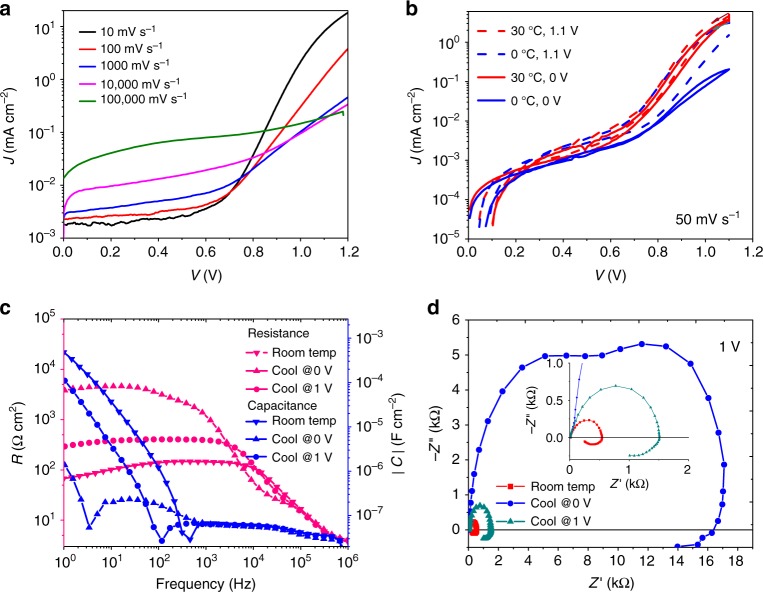
*JV* curves for negative-capacitance device. **a** Logarithmic representation of the forward scan (0–1.2 V) of Fig. 5a, indicating an increased series resistance for faster scans. **b** *JV* curves (50 mV s^−1^) measured after equilibration at 0 V (solid) and 1.1 V (dashed), at 30 °C (red) and 0 °C (blue), showing a higher series resistance for lower temperatures. **c** and **d** EIS data obtained at different temperatures (cool means 0 °C). Cooling-down has been done while keeping the device either at 0 or 1 V to freeze different ion distributions. Cooling down at 0 V induces a higher resistance consistent with the *JV* data

Examining the logarithmic plots of the *JV* curves (forward scan) for the negative-capacitance device from Fig. 5a in  Fig. 7a, we cannot observe a shift of the exponential region. Instead, the overall slope decreases for voltages larger than 0.8 V with enhanced scan rate. Thus, it is mainly a phenomenological series resistance that limits the forward current for faster scans rather than a changed recombination current. A possible explanation is that displaced ions that accumulate at the interface of the charge-transport layers introduce a thin space-charge layer^31^ (Debye layer^27^) or a dipole that decreases barriers for charge injection, as suggested for inverted hysteresis^32^, or the transient photomultiplication effect in perovskite photodetectors^33^. A similar effect of ions accumulating at interfaces that modify charge injection was proposed for barium titanate devices^34^, by a modeling study on organic solar cells^35^, and recently by a model of ionically gated transistor-like interfaces^16^.

We use temperature-dependent *JV* data (Fig. 7b) to further confirm that changed charge injection properties are responsible for the apparent negative capacitance, rather than modified recombination. This approach can be motivated by the following considerations:

1 $$J = J_{\mathrm{S}}\left( {{\mathrm{exp}}\frac{{eV}}{{nk_{\mathrm{B}}T}} - 1} \right) \Rightarrow R^{ - 1} = \frac{{{\mathrm{d}}J}}{{{\mathrm{d}}V}} \propto {\mathrm{exp}}\frac{{eV}}{{nk_{\mathrm{B}}T}}.$$

2 $$J \propto {\mathrm{exp}}\left( { - \frac{{E_{\mathrm{A}}\left( V \right)}}{{k_{\mathrm{B}}T}}} \right) \Rightarrow R^{ - 1} = \frac{{{\mathrm{d}}J}}{{{\mathrm{d}}V}} \propto {\mathrm{exp}}\left( { - \frac{{E_{\mathrm{A}}\left( V \right)}}{{k_{\mathrm{B}}T}}} \right).$$

The first equation (Shockley diode) describes recombination, which predicts a reduction of *R* with decreased *T* as e.g. observed in ref. ^36^. In contrast, the second equation describing temperature-activated charge-carrier injection over a barrier with energy *E*_A_ leads to an increase of *R* for lower *T*. Here, *E*_A_ can be expressed e.g. by thermionic emission yielding a decrease of *E*_A_ with applied voltages. Thus, examining the trend of *R* with *T* and preconditioning voltage should be sufficient to discriminate recombination from injection.

**Fig. 8:**
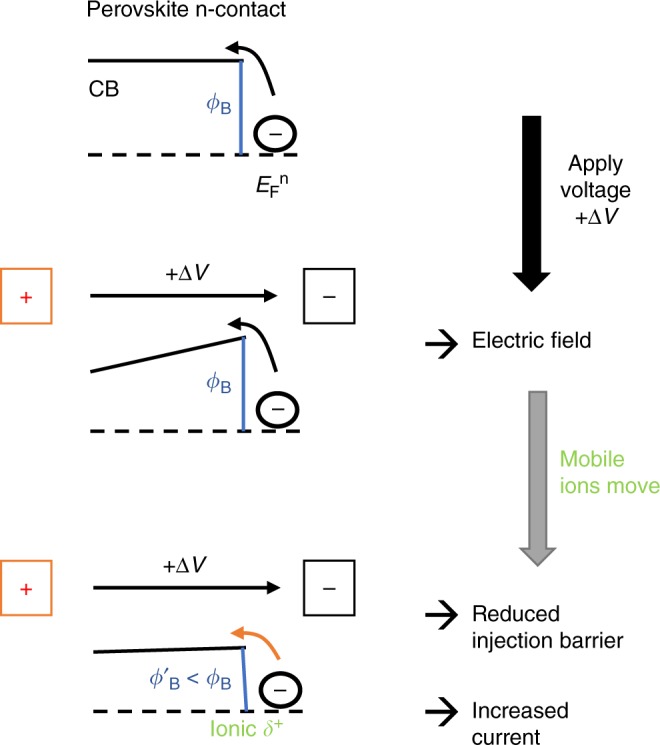
Sketch explaining ion-enhanced charge-carrier injection at the contact. Here the n-side is shown. An analogous picture can be drawn for the p-side. Ion redistribution upon increasing the voltage by Δ*V* changes the injection barrier *ϕ*_B_. More positive net ionic charge leads to a surface dipole that decreases *ϕ*_B_. This effect enhances the current, which appears as a negative capacitance

First we perform *JV* scans at 30 °C from 0 V (red-solid line in Fig. 7b) and from 1.1 V (red dashed) after having let the device equilibrate for 20 s at the respective starting voltage. Due to the moderate scan rate of 50 mV s^−1^ we observe similar *JV* curves with only weak hysteresis and a clear exponential region between 0.8 and 1 V. Then we cool down to 0 °C while applying either 0 V or 1.1 V to freeze the respective ion profiles. The subsequently measured *JV* curves (solid and dashed blue) do not coincide as the thermal energy is not sufficient to change ion profiles during the scan. Both curves show a much stronger series resistance effect. Interestingly, also the curve starting from 1.2 V (blue dashed) does not exhibit the expected change for a diode curve, i.e. a steeper and ‘right-shifted’ exponential region^36^ (Eq. 1), but a reduced slope, characteristic for an increased phenomenological series resistance as predicted by Eq. 2. The fact that freezing at different voltages results in different strength of hysteresis might be indicative of a ‘stabilization’ effect for interface charge^37^ dependent on their distribution. To further quantify this resistance, we performed EIS measurements at 1 V (Fig. 7c, d). Also there we see that cooling increases the resistance. Furthermore, cooling at 0 V leads to the highest resistance (which is not the case for a device without negative capacitance, Supplementary Fig. 12). This is consistent with ion distributions depending on voltage. The more negative (positive) the charge is at the electron (hole) injecting interface, the higher the barrier for injection. Lower applied voltages favor this distribution of mobile ions, which tend to screen the field in the perovskite film. Higher applied voltages, instead, lead to a more positive (negative) charge at the electron (hole) injecting interface (Fig. 8). The resulting surface charge decreases the injection barrier. *C*_high *f*_ is independent of *T* (Fig. 7c), as expected from a geometrical capacitance. The associated *τ*_high *f*_ decreases roughly by a factor of 2 with *T* due to the higher resistance. In contrast, the slow process leading to the apparent negative capacitance is roughly 10 times slower, consistent with temperature-activated ion migration.

We conclude that the *JV* characteristics in forward bias are highly dominated by temperature-activated transport of charges, presumably in, and from charge-transport layers into the perovskite. Therefore, the negative capacitance is indeed related to charge injection from the contacts. This explains why negative capacitance is often observed dependent on the charge-transport layers (mostly ETL).

Eqs (1) and (2) provide qualitative insights into changes of the injection current upon ion redistribution. In the following we sketch a semi-quantitative model on how the apparent capacitance is influenced by this process. We discuss the problem again in the time domain:

We assume that a step in voltage Δ*V* leads to a capacitive ionic response, in the simplest case with a single time constant *τ*_ion_ = *R*_ion_*C*_ion_:

3 $$J_{{\mathrm{ion}}}\left( t \right) = J_0e^{ - \frac{t}{{\tau _{{\mathrm{ion}}}}}}.$$

This current flowing through *R*_ion_  charges a capacitor by forming a Debye layer (couple of nm wide)^27^ at the interface between the perovskite layer and the charge-transport layer. *J*_0_ is the current at time 0, which equals *J*_0_ = Δ*V*/*R*_ion_, as the capacitor behaves like a short circuit while switching the voltage. The additional charge will be:

4 $${\mathrm{\Delta }}Q_{{\mathrm{ion}}}\left( t \right) = \int_0^t J_{{\mathrm{ion}}}{\rm{d}}t \prime = - J_0\tau _{{\mathrm{ion}}}\left( {e^{ - \frac{t}{{\tau _{{\mathrm{ion}}}}}} - 1} \right).$$

The opposite charge appears on the charge-transport layer leading to a change of the voltage drop over this capacitor ${\mathrm{\Delta }}U(t) = \frac{{{\mathrm{\Delta }}Q_{{\mathrm{ion}}}\left( t \right)}}{C}.$ As already mentioned above, this change in voltage will influence charge-carrier injection. As details on the charge-carrier injection mechanism into perovskite films are not (yet) well understood, we use the general thermionic emission equation (Richardson-Schottky), which is consistent with Eq. (2):

5 $$J_{{\mathrm{el}}} = A^ \ast T^2{\mathrm{exp}}\left( { - \frac{{\phi _{\mathrm{B}} - \sqrt {\frac{{e^3E}}{{4\pi {\it{\epsilon }}}}} }}{{k_BT}}} \right),$$

where A* is the Richardson constant and *ϕ*_B_ the injection barrier (Fig. 8). The second term in the exponent describes a lowering of the barrier by the electric field *E*. Accumulated ionic charge can influence both the electric field *E* and the barrier *ϕ*_B_. Assuming that the piled-up charge can be treated as a surface dipole, we consider that it changes the barrier:

6 $${\mathrm{\Delta }}\phi _{\mathrm{B}}(t) = e{\mathrm{\Delta }}U\left( t \right).$$

Expressing Δ*ϕ*_*B*_ with Eq. (4) and inserting it in Eq. (5), we find that the time constant is maintained and the change in injection current should follow:

7 $${\mathrm{\Delta }}J_{{\mathrm{el}}}\left( t \right) \propto \left\{ {\begin{array}{*{20}{l}} {\frac{{{\mathrm{\Delta }}V}}{{R_{{\mathrm{el}}}}}e^{ - \frac{t}{{\tau _{{\mathrm{ion}}}}}}} \hfill & {{\mathrm{positive}}\,{\mathrm{capacitance}}} \hfill \\ {\frac{{{\mathrm{\Delta }}V}}{{R_{{\mathrm{el}}}}}\left( {1 - e^{ - \frac{t}{{\tau _{{\mathrm{ion}}}}}}} \right)} \hfill & {{\mathrm{negative}}\,{\mathrm{capacitance}}} \hfill \end{array}} \right..$$

Note that *R*_el_ does not equal *R*_ion_ but is a function of the parameters shown in Eq. (5) such as A* etc. When performing impedance measurements under forward bias, mainly this electronic current is measured, where the time constant is related to ion migration; whereas, the resistance is related to the electric current and *C* is a phenomenological capacitance, which can be predicted from *R*_el_ and *τ*_ion_. This model explains why *R*_high *f*_ and *R*_low *f*_ scale similarly with light intensity or voltage. Note that this derivation based on Eq. (5) is just an illustrative example. A more comprehensive model in the frequency domain can be found in a very recent work^16^.

In case of Bismuth impurities, the injection current sets in at lower voltages leading to observation of negative capacitance already at lower voltages  (Supplementary Fig. 4). Besides Bi acting as a defect, the reasons for this reduction in voltage might be related to enhanced surface recombination due to different energetics at the interface of charge-transport layer and perovskite, which would directly confirm our hypothesis of modified charge-carrier injection barriers. Indeed, a shift of work function towards higher values has been reported for Bi containing devices^38^, which could lead to a higher electron injection barrier at the FTO/TiO_2_ contact and a lower built-in voltage. The negative capacitance, which we can observe for SnO_2_-based devices, fits into this explanation scheme as well, because the conduction band of SnO_2_ is lower than that of TiO_2_^39^, potentially causing an injection barrier for electrons into the conduction band of the perovskite. This explanation rationalizes also the observed correlation with hysteresis (further examples in Supplementary Fig. 13 and Supplementary Table 2), where developing a comprehensive quantitative model is beyond the scope of this study.

In conclusion, we have shown that high (positive and negative) apparent capacitances for low frequencies in perovskite solar cells are not due to a classical capacitive effect but due to slow transients in the injection current. Consequently, their appearance correlates with hysteresis in current–voltage curves also measured in the dark. Therefore, these capacitances cannot be used to quantify charge accumulation. They are a phenomenological consequence of the assignment of an *RC* element to the time constant of the slow transient process. The respective resistances, however, are related to electron and hole injection, transport, and recombination in the solar cell, being mainly determined by the most limiting process.

## Methods

### Fabrication

Fluorine-doped tin oxide (FTO) glass sheet was etched with zinc powder and diluted HCl. Then the etched substrate was cleaned in an ultrasonic bath with 2% Hellmanex water solution, deionized water, acetone, and ethanol. The compact TiO_2_ was coated by spray pyrolysis from a precursor solution of titanium diisopropoxide bis(acetylacetonate) in anhydrous ethanol, followed by a layer of mesoporous TiO_2_. Perovskite solution was prepared by dissolving perovskite precursors in DMF:DMSO 4:1 (v:v) to obtain 1.35 M perovskite solution with composition of Cs_0.1_FA_0.9_Pb(Br_0.1_I_0.9_)_3_. Bi(III) iodide (Sigma-Aldrich, 99.99%) solution was prepared separately by dissolving BiI_3_ in DMF (0.31 M), which was used to prepare 0.2% Bi containing perovskite solution and the other concentrations were prepared by sequential dilution with pure perovskite solution. The perovskite film was spin coated on top of the TiO_2_ in a two-step program at 1000 and 6000 rpm for 10 and 20 s, respectively, in a glove box with nitrogen atmosphere. During the second step, 200 μL of chlorobenzene were poured on the spinning substrate after 15 s from the starting point of the second step. The hole transport layer was deposited by spin coating at 4000 rpm for 30 s, using a solution prepared by dissolving 72.3 mg Spiro-OMeTAD, 28.8 μL 4-tert-butylpyridine, and 17.5 μL of LiTFSI solution (520 mg/mL LiTFSI (98%, Merck) in acetonitrile) in 1 mL chlorobenzene. As the last layer, the gold top electrode was thermally deposited under high vacuum. For planar devices, TiO_2_ was replaced by SnO_2_ deposited by ALD^39^.

### Characterization

Dark *JV* curves and impedance spectroscopy measurements were carried out by using a Biologic potentiostat (model SP-300). Transient current curves were measured using a Tektronix Oscilloscope (model DPO 7104C Digital phosphor oscilloscope) and Tektronix function generator (model AFG3022C) was used to apply square shape voltage to the cells. We used a white LED as the light source for impedance measurements under light and the incident light intensity was calibrated with measurements under a solar simulator (450 W Xenon light source, Oriel) to generate the same current density as under 1 sun illumination. Whereas all the measurements at room temperature were performed under ambient atmosphere with dry-air flow, the temperature-dependent measurements were done under N_2_ atmosphere in a home-built Peltier cooled sample holder.

## Supplementary information

Supplementary Information

## Data Availability

The data contained in Supporting Information are accessible through embedded objects. All the data are available from the authors.

## References

[1] Kern R, Sastrawan R, Ferber J, Stangl R, Luther J (2002). Modeling and interpretation of electrical impedance spectra of dye solar cells operated under open-circuit conditions. Electrochim. Acta.

[2] Fabregat-Santiago F (2007). Correlation between photovoltaic performance and impedance spectroscopy of dye-sensitized solar cells based on ionic liquids. J. Phys. Chem. C.

[3] Bisquert J, Garcia-Belmonte G, Pitarch Aacute, Bolink HJ (2006). Negative capacitance caused by electron injection through interfacial states in organic light-emitting diodes. Chem. Phys. Lett..

[4] Ehrenfreund E, Lungenschmied C, Dennler G, Neugebauer H, Sariciftci NS (2007). Negative capacitance in organic semiconductor devices: bipolar injection and charge recombination mechanism. Appl. Phys. Lett..

[5] Mora-Seró I (2006). Implications of the negative capacitance observed at forward bias in nanocomposite and polycrystalline solar cells. Nano Lett..

[6] Barna A, Horelick D (1971). A simple diode model including conductivity modulation. IEEE Trans. Circuit Theory.

[7] Bisquert J (2011). A variable series resistance mechanism to explain the negative capacitance observed in impedance spectroscopy measurements of nanostructured solar cells. Phys. Chem. Chem. Phys..

[8] Knapp E, Ruhstaller B (2015). Analysis of negative capacitance and self-heating in organic semiconductor devices. J. Appl. Phys..

[9] Shimizu K, Tanaka Y, Noguchi Y, Ishii H (2017). Negative capacitance in an organic solar cell observed by displacement current measurement. J. Phys. Conf. Ser..

[10] Dualeh A (2014). Impedance spectroscopic analysis of lead iodide perovskite-sensitized solid-state solar cells. ACS Nano.

[11] Zohar A (2016). Impedance spectroscopic indication for solid state electrochemical reaction in (CH_3_NH_3_)PbI_3_ films. J. Phys. Chem. Lett..

[12] Fabregat-Santiago F (2017). Deleterious effect of negative capacitance on the performance of halide perovskite solar cells. ACS Energy Lett..

[13] Guerrero A (2016). Properties of contact and bulk impedances in hybrid lead halide perovskite solar cells including inductive loop elements. J. Phys. Chem. C.

[14] Feng Y (2018). Interfacial negative capacitance in planar perovskite solar cells: an interpretation based on band theory. Mater. Res. Bull..

[15] Jacobs DA (2018). The two faces of capacitance: new interpretations for electrical impedance measurements of perovskite solar cells and their relation to hysteresis. J. Appl. Phys..

[16] Moia, D. et al. Ionic-to-electronic current amplification in hybrid perovskite solar cells. Preprint at http://arxiv.org/abs/1805.06446 (2018).

[17] Juarez-Perez EJ (2014). Photoinduced giant dielectric constant in lead halide perovskite solar cells. J. Phys. Chem. Lett..

[18] Zarazua I, Bisquert J, Garcia-Belmonte G (2016). Light-induced space-charge accumulation zone as photovoltaic mechanism in perovskite solar cells. J. Phys. Chem. Lett..

[19] Tress W (2015). Understanding the rate-dependent J–V hysteresis, slow time component, and aging in CH_3_NH_3_PbI_3_ perovskite solar cells: the role of a compensated electric field. Energy Environ. Sci..

[20] Pockett A (2017). Microseconds, milliseconds and seconds: deconvoluting the dynamic behaviour of planar perovskite solar cells. Phys. Chem. Chem. Phys..

[21] Nayak PK (2018). Impact of Bi3+ heterovalent doping in organic–inorganic metal halide perovskite crystals. J. Am. Chem. Soc..

[22] Zarazua I (2016). Surface recombination and collection efficiency in perovskite solar cells from impedance analysis. J. Phys. Chem. Lett..

[23] Ghahremanirad E, Bou A, Olyaee S, Bisquert J (2017). Inductive loop in the impedance response of perovskite solar cells explained by surface polarization model. J. Phys. Chem. Lett..

[24] Yang Q (2018). Surface polarization and recombination in organic-inorganic hybrid perovskite solar cells based on photo- and electrically induced negative capacitance studies. Org. Electron..

[25] Ravishankar S (2017). Surface polarization model for the dynamic hysteresis of perovskite solar cells. J. Phys. Chem. Lett..

[26] Tress W (2017). Metal halide perovskites as mixed electronic–ionic conductors: challenges and opportunities—from hysteresis to memristivity. J. Phys. Chem. Lett..

[27] Richardson G (2016). Can slow-moving ions explain hysteresis in the current–voltage curves of perovskite solar cells?. Energy Environ. Sci..

[28] Ershov M (1998). Negative capacitance effect in semiconductor devices. IEEE Trans. Electron Devices.

[29] Yang TY, Gregori G, Pellet N, Grätzel M, Maier J (2015). The significance of ion conduction in a hybrid organic–inorganic lead-iodide-based perovskite photosensitizer. Angew. Chem..

[30] Kim GY (2018). Large tunable photoeffect on ion conduction in halide perovskites and implications for photodecomposition. Nat. Mater..

[31] Neukom MT (2017). Why perovskite solar cells with high efficiency show small IV-curve hysteresis. Sol. Energy Mater. Sol. Cells.

[32] Tress W, Correa Baena JP, Saliba M, Abate A, Graetzel M (2016). Inverted current–voltage hysteresis in mixed perovskite solar cells: polarization, energy barriers, and defect recombination. Adv. Energy Mater..

[33] Domanski K (2015). Working principles of perovskite photodetectors: analyzing the interplay between photoconductivity and voltage-driven energy-level alignment. Adv. Funct. Mater..

[34] El Kamel, F., Gonon, P., Jomni, F. & Yangui, B. Observation of negative capacitances in metal-insulator-metal devices based on a-BaTiO3:H.* Appl. Phys. Lett.* **93**, 042904 (2008).

[35] Wang, X. Q. & Cai, C. B. A possible mechanism for the negative capacitance observed in organic devices. Preprint at http://arxiv.org/abs/1210.7904 (2012).

[36] Tress W (2018). Interpretation and evolution of open-circuit voltage, recombination, ideality factor and subgap defect states during reversible light-soaking and irreversible degradation of perovskite solar cells. Energy Environ. Sci..

[37] Weber SAL (2018). How the formation of interfacial charge causes hysteresis in perovskite solar cells. Energy Environ. Sci..

[38] Yavari, M. et al. How far does the defect tolerance of lead-halide perovskites range? The example of Bi impurities introducing efficient recombination centers. *J. Mater. Chem. A.*10.1039/C9TA01744E (2019).

[39] Baena JPC (2015). Highly efficient planar perovskite solar cells through band alignment engineering. Energy Environ. Sci..

