# Supplementary material for: Origin of apparent light-enhanced and negative capacitance in perovskite solar cells
Source: Nat Commun. 2019 Apr 5;10:1574. doi: 10.1038/s41467-019-09079-z (PMC6450882; doi:10.1038/s41467-019-09079-z)
Supplement: Supplementary file 1 — Supplementary Information [file 41467_2019_9079_MOESM1_ESM.pdf]

Origin of Apparent Light-Enhanced and Negative Capacitance in Perovskite Solar Cells

Firouzeh Ebadi[1,2], Nima Taghavinia[2,3*], Raheleh Mohammadpour[2], Anders Hagfeldt[1], Wolfgang Tress[1]*

[1]École Polytechnique Fédérale de Lausanne, Laboratory of Photomolecular Science, 1015 Lausanne, Switzerland

[2]Institute for Nanoscience and Nanotechnology, Sharif University of Technology, Tehran 14588, Iran

[3]Physics Department, Sharif University of Technology, Tehran 14588, Iran

Supplementary Information

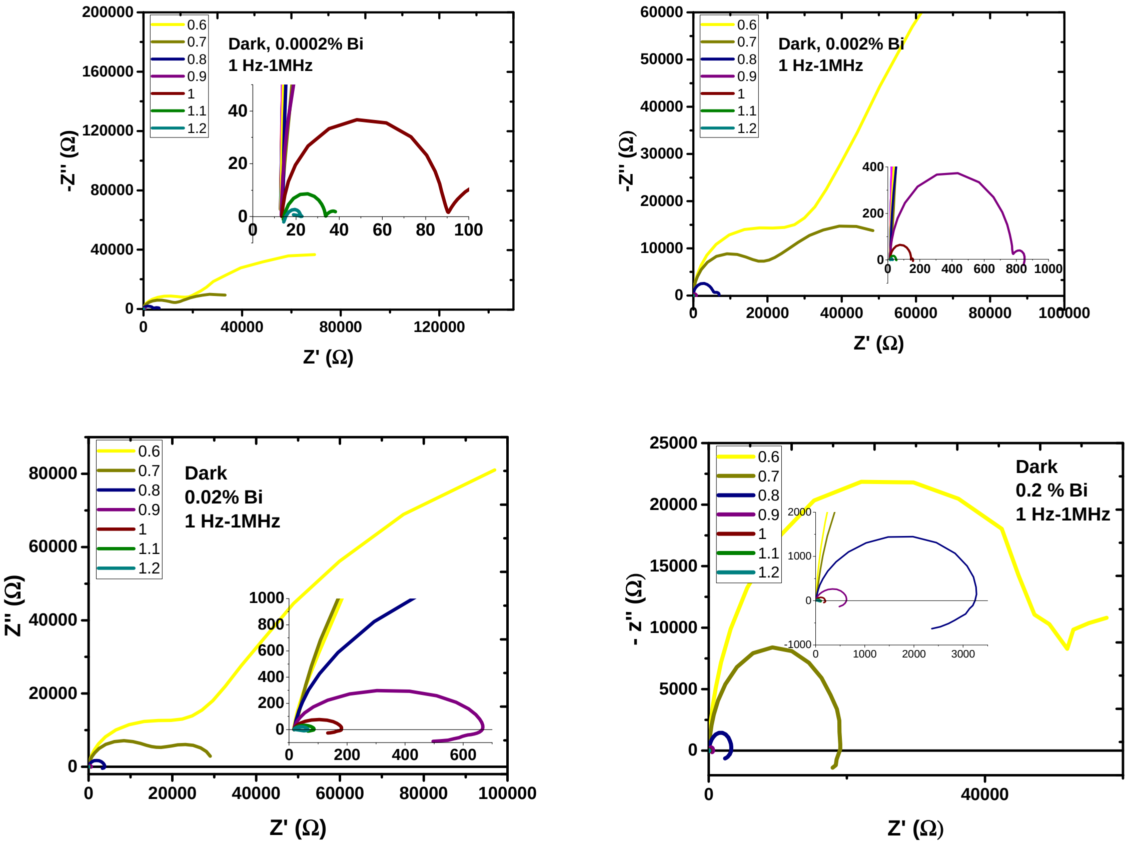

Supplementary Figure 1. Nyquist plots for devices with **different Bi concentrations** measured at different voltages (values in legend in V) showing appearance of negative capacitance for higher voltages and systematically for higher Bi concentrations.

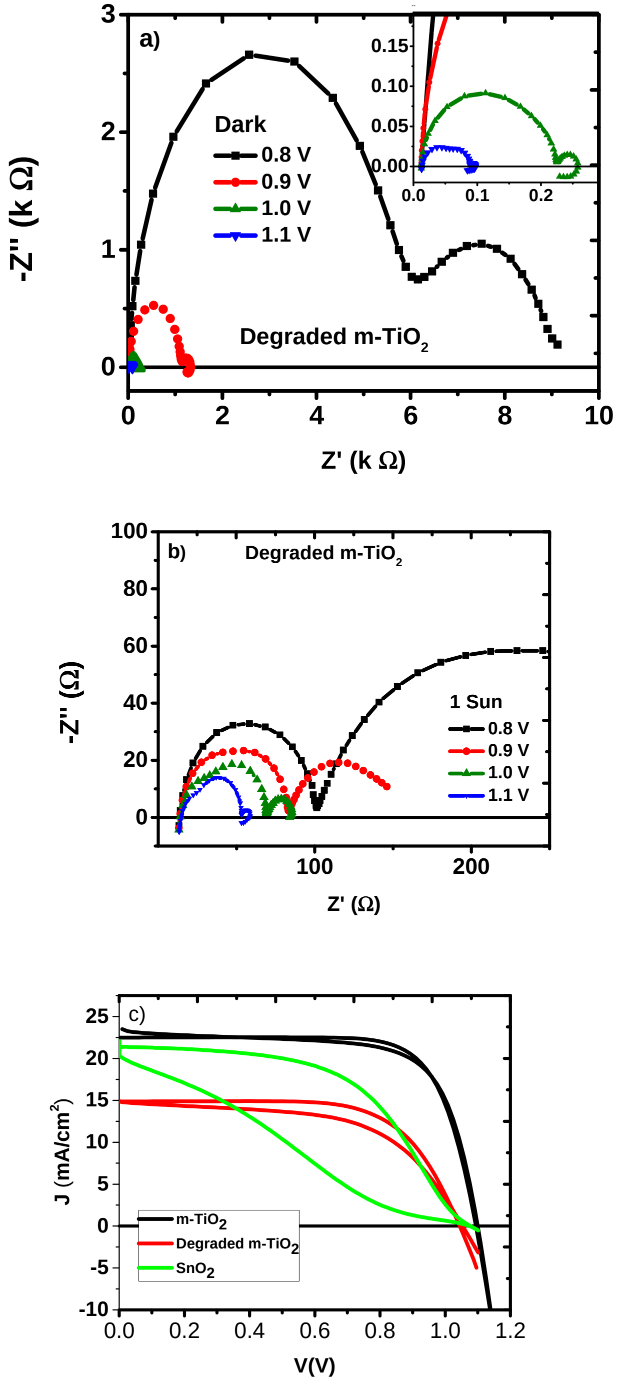

Supplementary Figure 2. Stored mesoporous TiO_2 based device (a and b) Nyquist plots in the dark and under 1 sun illumination. (c) *JV* curve under 1 sun compared to pristine TiO_2 and SnO_2 devices measured under 100 mV s^{-1}.

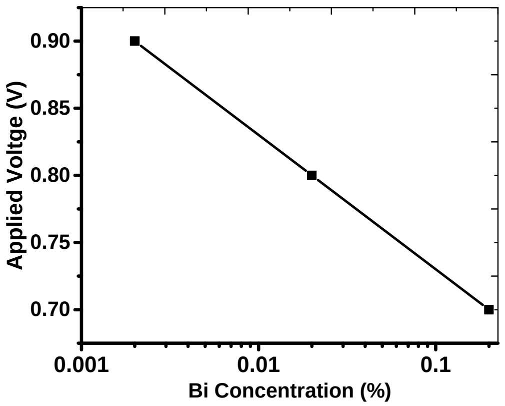

Supplementary Figure 3. Voltage, where the reactance changes sign and the apparent capacitance becomes negative as a function of **Bi concentration**. Negative capacitance becomes apparent for lower voltages for higher Bi concentration.

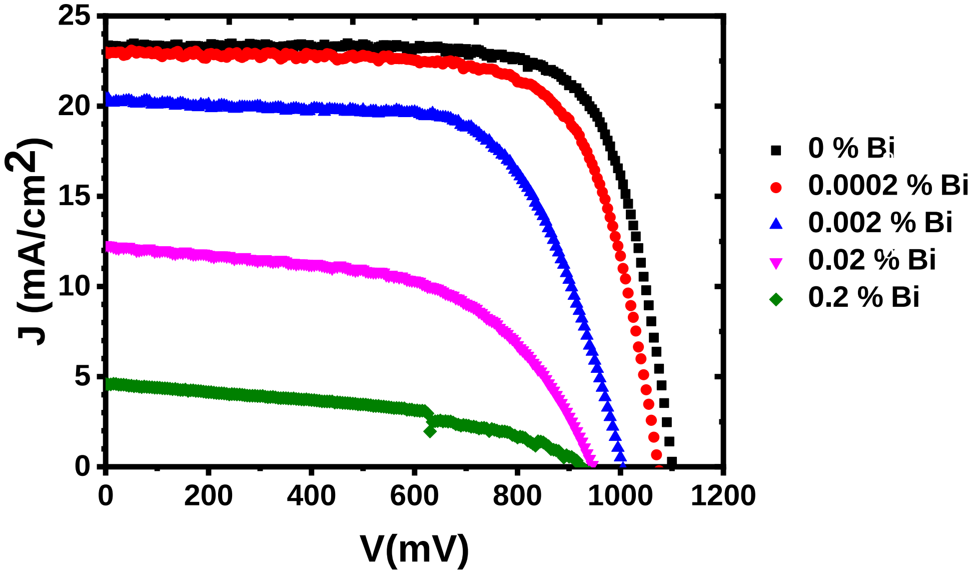

Supplementary Figure 4. Representative *JV* curves for the Bi series, obtained from a backward scan with scan rate of 100 mV s^{-1}.

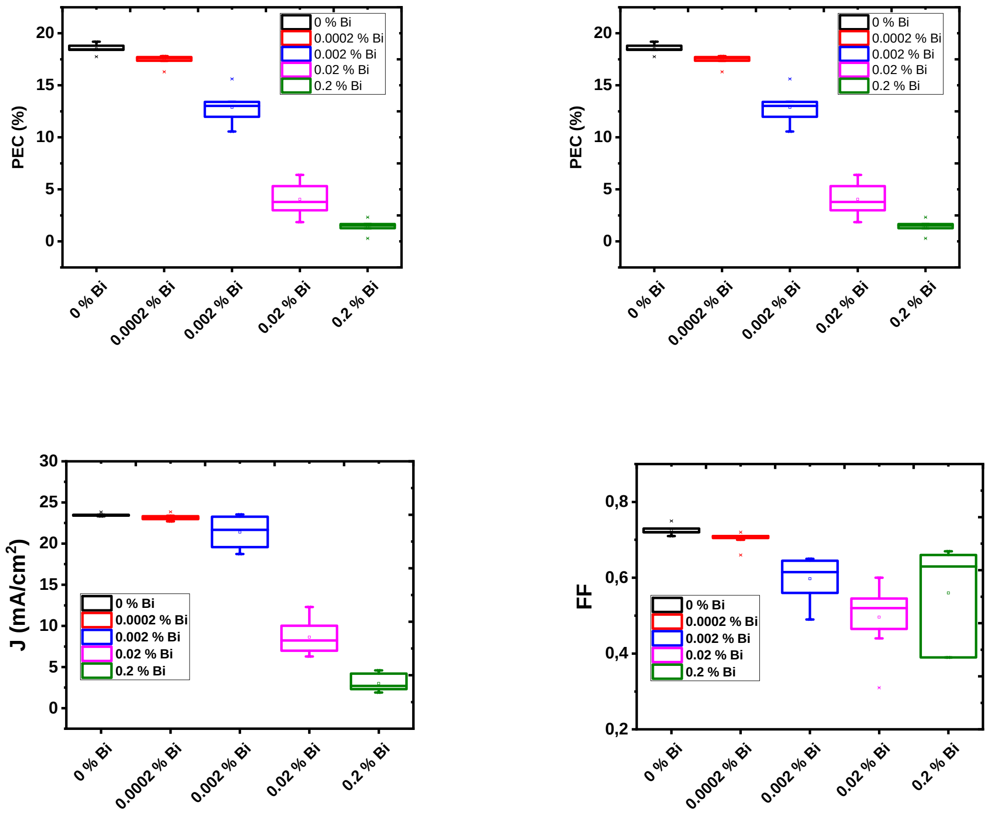

Supplementary Figure 5. *JV* parameters for the Bi series obtained from backwards scan with a scan rate of 100 mV s^{-1}.

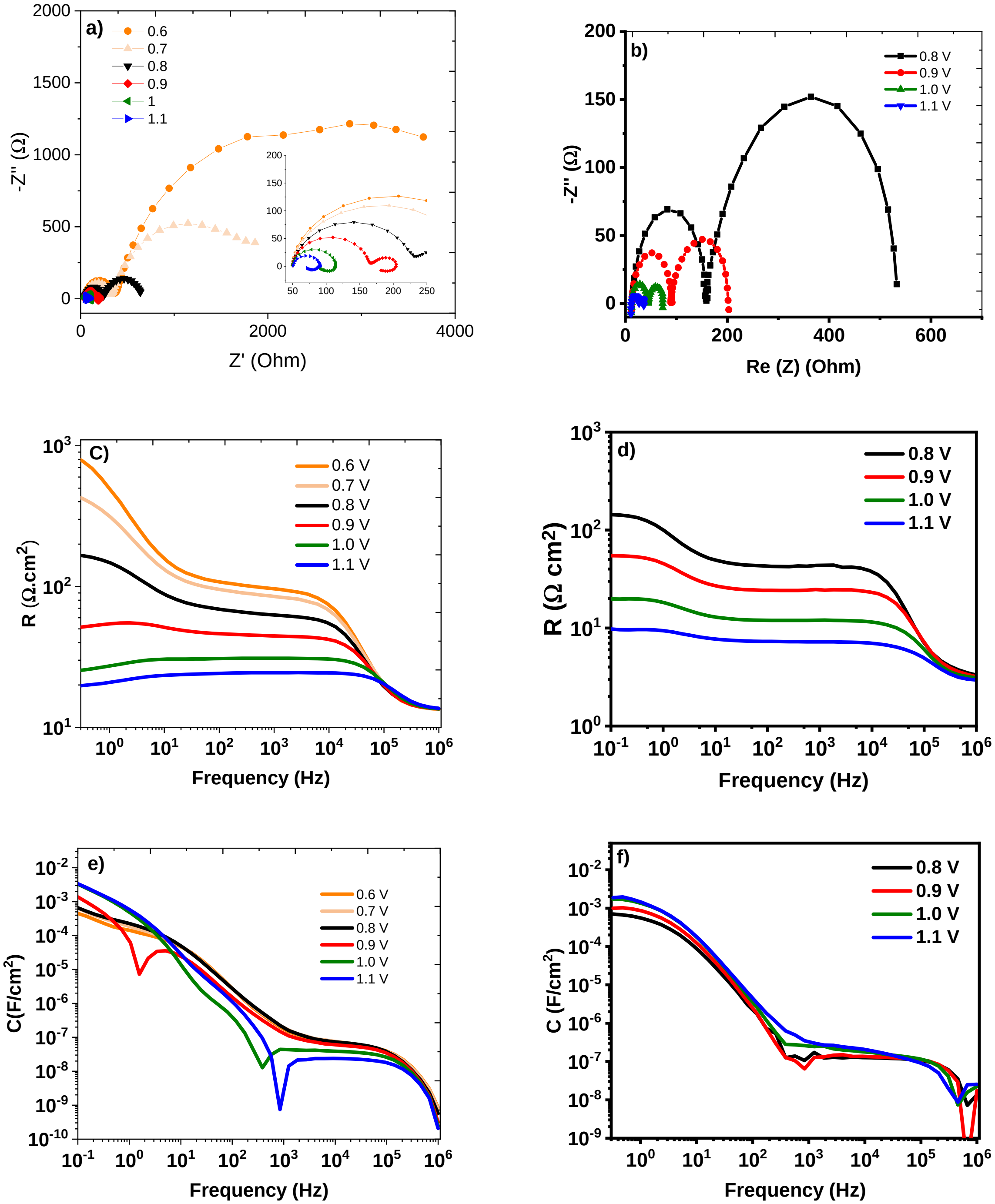

Supplementary Figure 6. EIS data as a function of applied voltage under illumination (1 sun) to complement Fig. 2 of the main manuscript.

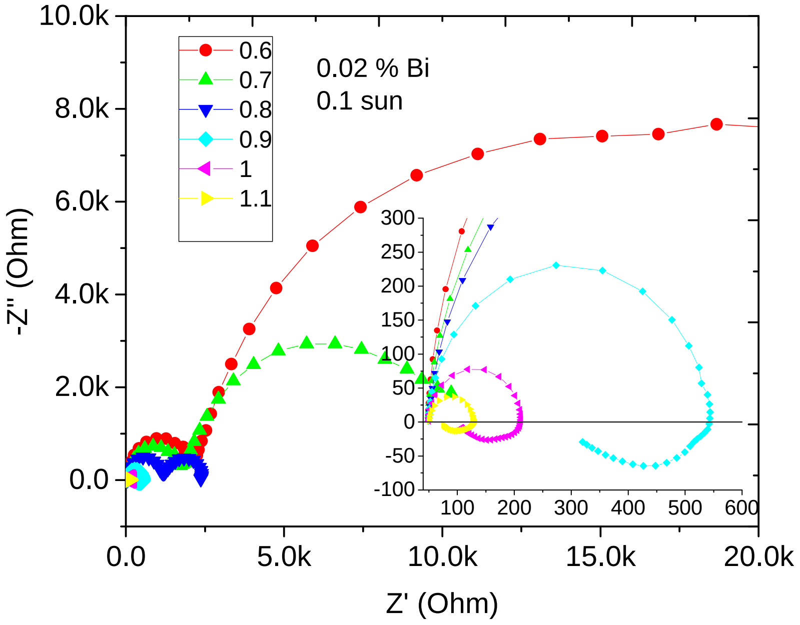

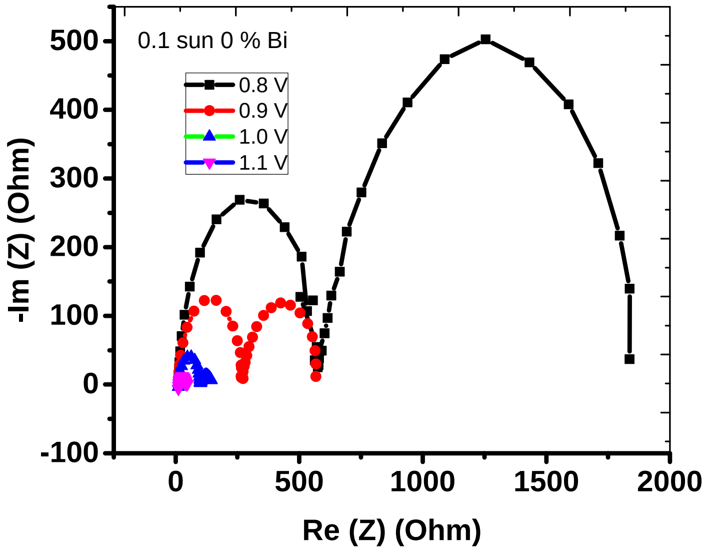

Supplementary Figure 7. Nyquist plots for **device with and without negative capacitance** under illumination (0.1 sun) to complement Fig. 2 and 3 of the main manuscript.

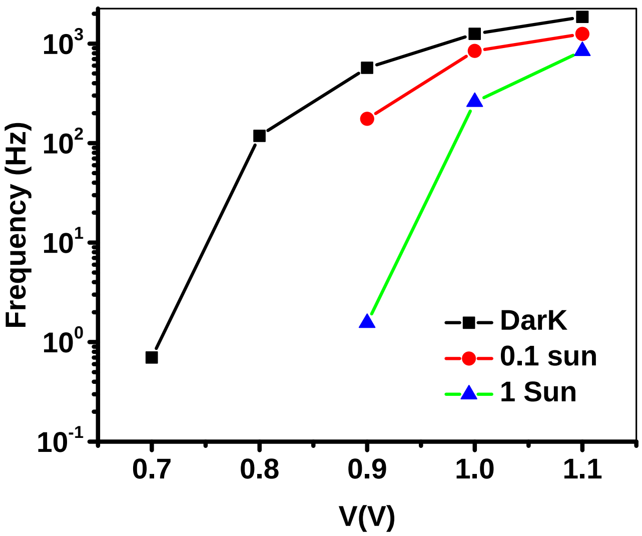

Supplementary Figure 8. Frequency, where the reactance changes sign and the apparent capacitance becomes negative as a function of voltage for different illumination intensities. Negative capacitance becomes apparent under forward conditions and more pronounced (i.e. sets in already at higher frequencies for higher voltages).

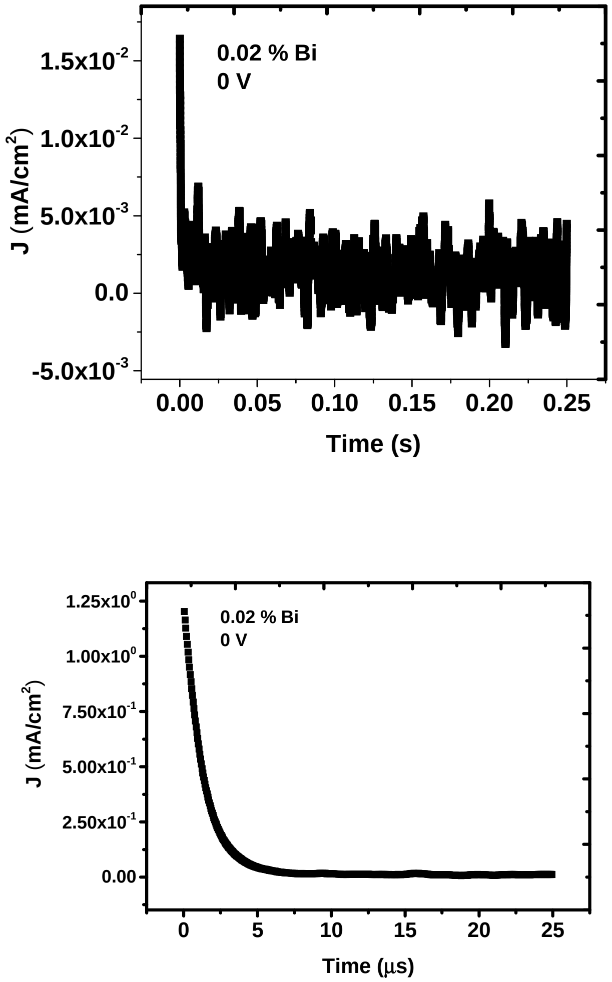

Supplementary Figure 9. Transient data measured for a **negative-capacitance device at 0 V**. Consistent with the missing negative capacitance in the EIS data, there is no transient on the long time scales. Only the μs-RC response, which is independent of applied voltage, can be seen in the zoom (bottom).

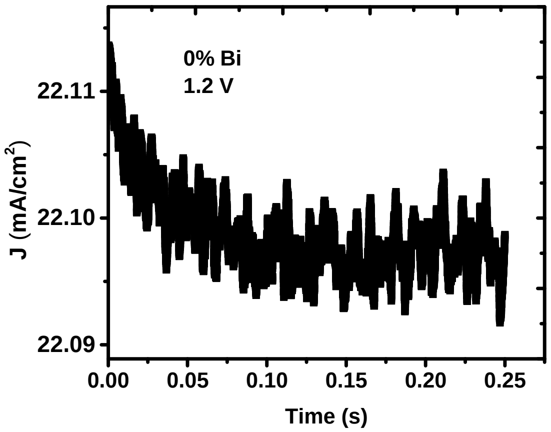

Supplementary Figure 10. Transient data measured for a **device without negative capacitance at 1.2 V**. The current is almost constant (changes are below 0.1%) with a slight decrease consistent with a positive low-frequency time constant observed in EIS.

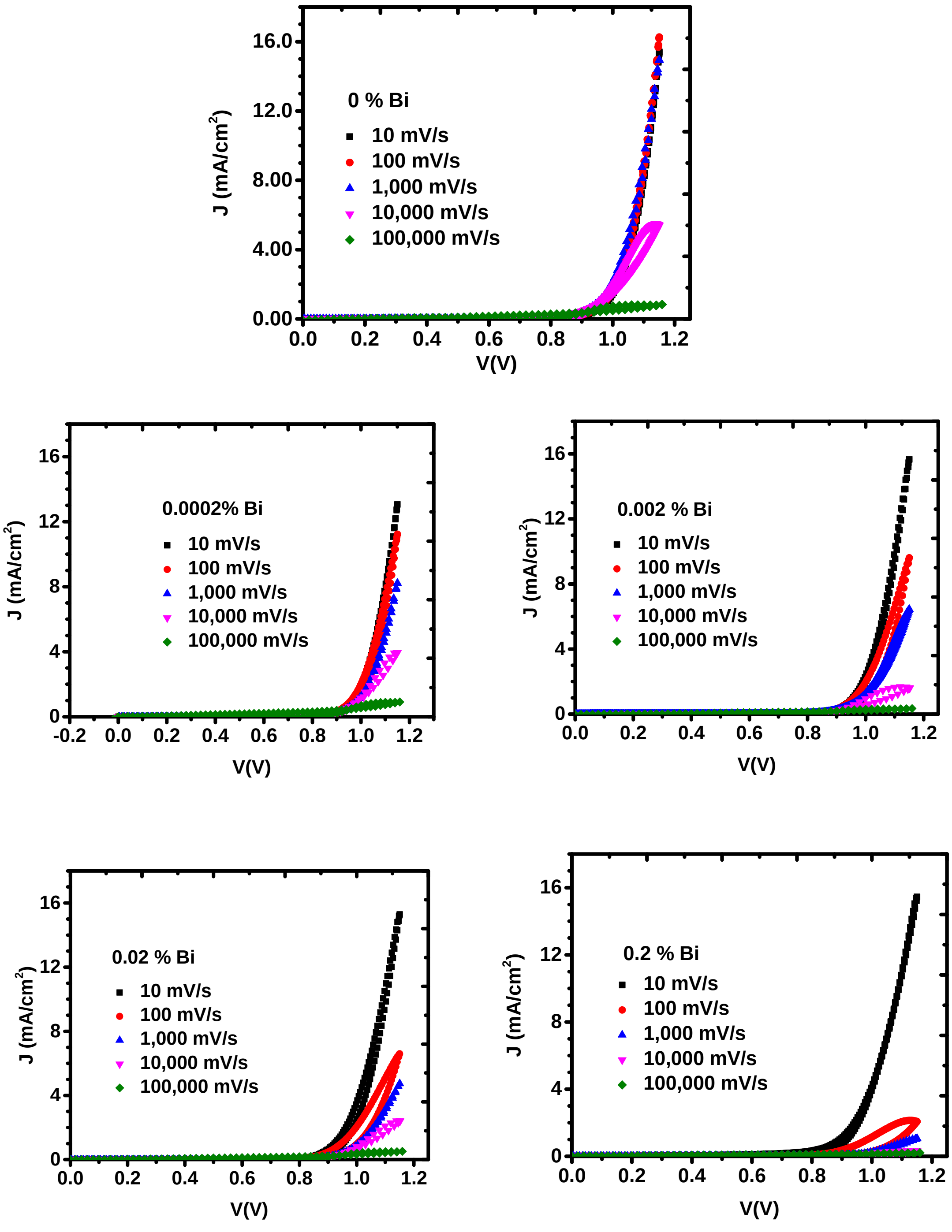

Supplementary Figure 11. Scan-rate dependent *JV* curves for the whole Bi series.

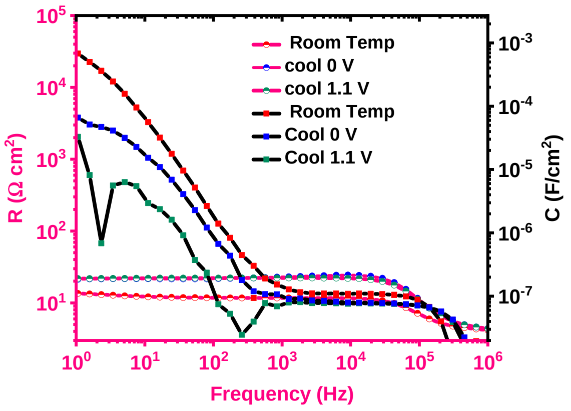

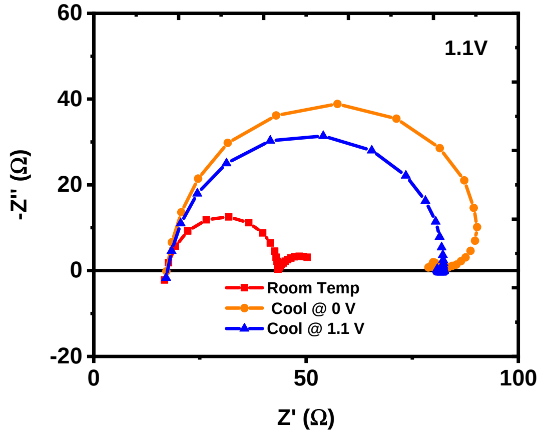

Supplementary Figure 12. Effect of temperature on device without negative capacitance. The resistance is hardly influenced by the voltage during cooling indicating that different ion distributions do not influence forward current considerably.

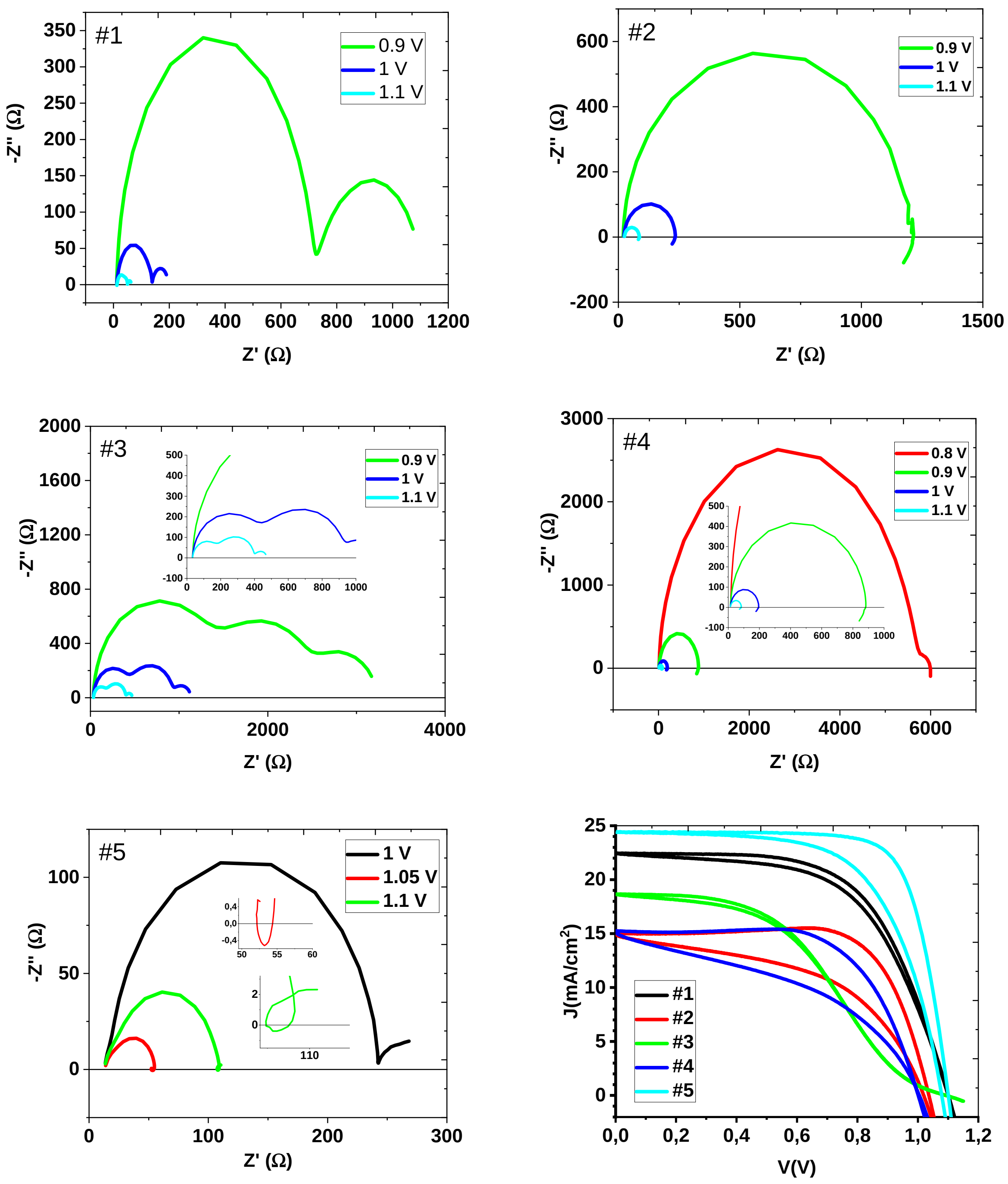

Supplementary Figure 13. EIS of 5 different devices in the dark and hysteresis *JV* curves (50 mV s^{-1}) under 1 sun illumination. A summary including hysteresis index is given in Supplementary Table 2 below.

Supplementary Table 1 Summary of the low-frequency time constants obtained from fitting the EIS (Fig. 3 of manuscript) and transient data for the negative capacitance device.

Dark (From EIS)	0.9 V	1 V	1.1 V
τ_1 (s)	0.003	0.003	0.003
τ_2 (s)	0.037	0.034	0.043
τ_3 (s)	0.520	0.460	0.423
**0.1 Sun (From EIS)**	**0.9 V**	**1 V**	**1.1 V**
τ_1 (s)	-	0.003	0.004
τ_2 (s)	0.09	0.04	0.040
τ_3 (s)	0.300	0.300	0.300
**1 Sun (From EIS)**	**0.9 V**	**1 V**	**1.1 V**
τ_1 (s)	-	0.002	0.003
τ_2 (s)	0.018	0.090	0.070
τ_3 (s)	0.620	0.510	0.361
**Dark (from Oscilloscope)**	**0.9 V**	**1 V**	**1.1 V**
τ_1 (s)	0.002	0.005	0.004
τ_2 (s)	0.032	0.040	0.037
τ_3 (s)	0.232	0.248	0.212

Supplementary Table 2 Summary of the data from Supplementary Figure 13. The hysteresis index is calculated as: $\frac{\int JV_{(\mathrm{backward})}\mathrm{d}V - \int JV_{(\mathrm{forward})}\mathrm{d}V}{\int JV_{(\mathrm{backward})}\mathrm{d}V + \int JV_{(\mathrm{forward})}\mathrm{d}V}$ according to Nemnes et al. Solar Energy **173**, 2018, Pages 976-983

Hysteresis Index	Neg-cap Voltage	Device performance
0.01	No neg-cap	Poor (#3)
0.02	No neg-cap	Good (#1)
0.04	1.05 V (Neg loop) 1.1 V Neg-cap	High efficient >20 (#5)
0.11	0.9 V	Poor (#2)
0.13	0.8 V	Poor (#4)
